# Downregulation of CD40L–CD40 attenuates seizure susceptibility and severity of seizures

**DOI:** 10.1038/s41598-021-96760-3

**Published:** 2021-08-26

**Authors:** Esther Pototskiy, Katherine Vinokuroff, Andrew Ojeda, C. Kendall Major, Deepak Sharma, Taylor Anderson, Kendall Howard, Ronen Borenstein, Alberto E. Musto

**Affiliations:** 1 Department of Pathology and Anatomy, Eastern Virginia Medical School, 700 W. Olney Road, Lewis Hall, Office 2174, Norfolk, VA 23507 USA; 2 Department of Neurology, Eastern Virginia Medical School, 700 W. Olney Road, Lewis Hall, Office 2174, Norfolk, VA 23507 USA; 3 Eastern Virginia Medical School, Norfolk, VA USA; 4 Department of Microbiology and Molecular Cell Biology, Eastern Virginia Medical School, Norfolk, VA 23507 USA

**Keywords:** Epilepsy, Epilepsy, Inflammation

## Abstract

Unregulated neuro-inflammation mediates seizures in temporal lobe epilepsy (TLE). Our aim was to determine the effect of CD40–CD40L activation in experimental seizures. CD40 deficient mice (CD40KO) and control mice (wild type, WT) received pentenyltetrazole (PTZ) or pilocarpine to evaluate seizures and status epilepticus (SE) respectively. In mice, anti-CD40L antibody was administered intranasally before PTZ. Brain samples from human TLE and post-seizure mice were processed to determine CD40–CD40L expression using histological and molecular techniques. CD40 expression was higher in hippocampus from human TLE and in cortical neurons and hippocampal neural terminals after experimental seizures. CD40–CD40L levels increased after seizures in the hippocampus and in the cortex. After SE, CD40L/CD40 levels increased in cortex and showed an upward trend in the hippocampus. CD40KO mice demonstrated reduction in seizure severity and in latency compared to WT mice. Anti-CD40L antibody limited seizure susceptibility and seizure severity. CD40L–CD40 interaction can serve as a target for an immuno-therapy for TLE.

## Introduction

Among the different types of epilepsies, Temporal Lobe Epilepsy (TLE) or limbic epilepsy is one of the most common forms of focal epilepsy in adults^1,2^. Although most patients with TLE respond favorably to current anti-epileptic drugs (AED), almost 30% of TLE patients have limbic seizures that become refractory to medical treatments, requiring surgical resection of parts of the temporal lobe. These patients have an increased risk of adverse drug reactions to AED, cognitive impairments, psychiatric comorbidities, or early mortality^3,4^. This implies that it is imperative to discover alternative pharmacotherapies to prevent recurrent seizures or modify the course of epilepsy.

TLE is associated with a history of previous brain injuries^1,5^ that trigger an unregulated molecular and cellular process in the neuronal network that participates in the initiation of seizures by inducing neuropathological changes mostly in hippocampal and in extra hippocampal regions such as the cortex^1,5^. Examples of such changes include neuronal hyperexcitability, interneuron damage, and aberrant post-synaptic formation^4^ which all contribute to a recurrent seizure state, a hallmark of epilepsy^1–7^.

Pro-inflammatory lipid mediators, cytokines, and chemokines mediate the initiation and maintenance of limbic seizures^8–11^ through diverse neuronal hyper-excitability mechanisms^10^. In addition, upregulation of neuro-inflammatory cellular mediators, such as IL-1, TNF, or PAF, may trigger epileptogenesis^10^ by inducing cellular damage and aberrant neuronal plasticity^6,9^.

CD40L–CD40 interactions have been implicated in inflammatory and immune responses^12–14^. CD40L, a small protein belonging to the TNF superfamily, interacts with its receptor, CD40, to mediate neurite organization during brain development^10,15^. This also acts as a link between the innate and adaptive immune system during times of imflammation due to cellular damage or dysregulation. CD40L–CD40 increases after stroke and status epilepticus and mediates the development of dystrophic neurites in Alzheimer’s disease^16–19^ and neuronal damage^20–22^. Since epilepsy research currently aims to understand the fundamental role of inflammation in seizure development, we studied the CD40L–CD40 activation in experimental seizures induced by pentylenetetrazol (PTZ) or pilocarpine in wild type (WT) and CD40 receptor-deficient adult mice (CD40KO) using animal models for translational clinical seizures. We observed that CD40L–CD40 expression increased in the brain after seizures. Also, we analyzed the effects of intranasal anti-CD40L antibody treatment against PTZ-induced seizures. We observed that either genetic deficiency of CD40 or intranasal administration of anti-CD40L antibody was able to limit seizure susceptibility, reducing the frequency of induced acute seizures. Therefore, targeting CD40–CD40L or their molecular signaling pathways could pave the way toward a new therapeutic approach against epilepsy.

## Materials and methods

Studies were performed according to the National Institutes of Health (NIH) guidelines and in accordance with nationally accepted principles in the care and use of experimental animals. The Institutional Animal Care and Use Committee at Eastern Virginia Medical School approved the animal protocol for this study (IACUC, #17-012). Water and food were available for ad libitum consumption in individual cages located at the EVMS Comparative Medicine Vivarium. Animals were fed a diet from ENVIGO containing a nutrient profile with 6.2% fat and 18.6% protein. All efforts were made to minimize pain and suffering and to reduce the number of mice used in these experiments. For euthanasia, animals were deeply exposed to isoflurane.

### Mice

Adult male mice (28–33 g) used included CD40 receptor-deficient knockout (B6.129P2-Tnfrsf5 tm1kitk, The Jackson Laboratory), and C57BL/6 as a control (WT) from The Jackson Laboratory. CD40KO mice were backcrossed to B6, and bought at ~ 13 weeks from Jackson Laboratories and used after arrival for experiments.

### PTZ model

After mice were placed in individual cages, pentylenetetrazol (PTZ a γ- aminobutyric acid subtype A (GABAA)-receptor antagonist, [10 mg/kg (six doses) or 75 mg/kg (one dose), dissolved in sterile saline, volume injected: 0.25 mL, intraperitoneal (IP)] (Sigma, St. Louis, MO, U.S.A.) was administered for induction of seizures. For seizure susceptibility, each mouse received injections every 5 min until the onset of the first retropulsive myoclonus, defined as a myoclonic jerk resulting in backward movement of the head and shoulders, and then subsequently euthanized. The latency to the onset of myoclonic jerk was the primary metric recorded. Seizures were classified according to the Racine scale^6^. (0: normal behavior—walking, exploring, sniffing, grooming; 1: immobile, staring, jumpy, curled-up posture; 2: automatisms—repetitive blinking, chewing, head bobbing, vibrissae twitching, scratching, face washing, “star gazing”; 3: partial-body clonus, occasional myoclonic jerks, shivering; 4: whole-body tonic-clonus, “corkscrew” turning and flipping, loss of posture, rearing, falling; 5: non-intermittent tonic–clonic seizure activity). Mice were observed continuously for at least 2 h with data recorded regarding time to achievement of respective Racine stage and duration of seizures.

### Immunohistology

Specimens from human TLE, sectioned and mounted in slides, coded, with no identifier and used only for research purposes were obtained from Dr. Tomokatsu Hori of the Department of Neurosurgery at Tokyo Women’s Medical University, Japan. Brains from mice were removed immediately after euthanization, fixed in formalin 4%, transferred in PBS, and followed by 30% sucrose. Coronal 30-µm-thick sections were collected for CD40 (CD40,1:500,sc-20010 Santa Cruz, Inc.) or co-localized with PSD-95 (PSD-95 Polyclonal Antibody Invitrogen Catalog # 51-6900 Concentration: 1:200) or GAP-43 (GAP-43 Novus Bio NB300-143 Concentration 1:200) in some sections for immunohistology. Mice brain tissue was collected after using Isoflurane for euthanasia. Each hemisphere was separated and purposed for either IHC or western blotting. The cerebral cortex and the hippocampus of one hemisphere were snap frozen and saved for western blotting. Half of the brain was purposed for IHC and submerged in 4% Paraformaldehyde (FD Neurotech, MD, USA) over a period of 7 days. After 7 days, samples were submerged in a 20% Sucrose solution in Phosphate-buffered saline (FD Neurotech, MD, USA) over 72 h. The Mouse/Rabbit Polydetector HRP/DAB kit was used (Mouse and Rabbit Specific HRP/DAB (ABC) Detection IHC kit (ab64264) or Alexa Fluor (Alexa Flour 488 Goat anti-rabbit Catalog # A-11008 concentration 1:1000; Goat anti-Rat IgG (H + L) Cross-Adsorbed Secondary Antibody, Alexa Fluor 594 (Catalog #: A-11007) Concentration 1:1000 in those section for co-localization studies. The sections were rinsed in water, dehydrated with ethanol, placed in xylene, mounted, and coverslipped. Cortex, dentate gyrus, CA1, and CA3 regions from the right hippocampus were examined by using standard light microscopy with a Zeiss imaging microscope system. Using a Leica CM1950 Cryostat, 20 micron-thick sections were cut and dried overnight. After slides were dry, they were rehydrated using Xylene and ethanol baths. Slides were treated with serum and other protein complexes to block nonspecific binding and were incubated overnight with multiple primary antibodies. Slides were washed with PBST and were incubated with a Horseradish Peroxidase conjugated secondary antibody. After at least an hour of the secondary incubation, slides were washed with PBST and were stained with 3,3′-Diaminobenzidine which developed the cell type isolated with the primary antibody. Imaging was performed using an Olympus microscope.

### Western blotting and ELISA

Samples that were snap frozen after dissection were placed in a -80 °C refrigerator until Western blotting was performed. To prepare the lysates, about 300 mg of tissue (cortex and hippocampus) was placed in 500uL of RIPA buffer. A homogenizer was used on the samples multiple times until the tissue disintegrated as much as possible. Samples were then agitated in an orbital shaker for 2 h at 4 °C. Samples were homogenized once more and then placed in a centrifuge at 4 °C and set to 12,000RPM for 20 min. The supernatant was then aliquoted. Bradford Assays were performed to quantify the protein concentration in each lysate. Using the Mini-Protean Tetra Cell assembly for SDS-PAGE (Bio-Rad, State, USA), along with Mini-Protean TGX 4–20% gels, samples were then loaded into their respective lanes, accounting for about 50ug of protein per lane. While analyzing CD40 and CD40L, the protein concentration was increased to about 100 ug, since CD40 and CD40L expression is expected to be low. Gels ran at 100 V over approximately an hour. Afterward, transfer to the nitrocellulose membrane was performed using a cassette set-up. The transfer was performed using 100 V over an hour. Nitrocellulose membranes after transfer were then hybridized with the primary antibody in a blocking solution that contained BSA and TBST, set overnight. Primary antibodies included CD40 (abcam.com, ab13545: 1:1000 concentration), CD40L (abcam.com, ab52750: 1:500 concentration), P38 (Invitrogen, PA5-17713: 1:1000 concentration), PP38 (Invitrogen, MA5-15177: 1:1000 concentration), and B-actin (Biolegend, Catalog # 622102: 1:1000 concentration). Membranes were then washed three times prior to secondary antibody hybridization and three more times after. Imaging was performed using the LicorOdyssey system (Li-Cor, State, USA). ELISA was followed by manufacture instructions (CD40L TNFSF5 ab119517, abcam.com).

### Synaptosome extraction

Brains were removed after euthanasia from control animals (saline IP) and from those with tonic–clonic seizures (Racine’s score > 3) following PTZ administration (75 mg/kg IP); then cortical and hippocampal regions were microdissected, and snap frozen at -80C. From those samples, synaptosomes were extracted using a tissue homogenizer and the Syn-PER Synaptic Protein Extraction Reagent (Thermo-Fisher Scientific Catalog # 87793) according to manufacturer’s protocol to extract synaptosome fractions from samples and controls. After the completion of the last centrifugal step, synaptosome pellets were re-suspended in the Syn-PER Synaptic Protein Extraction Reagent at around a concentration of 1 ml per 400 mg of tissue. Then CD40L and CD40 concentration was measured using a CD40L ELISA (ab119517, abcam.com) kit and a CD40 ELISA (ab100674, abcam.com) kit respectively. A separate protein curve was generated for each ELISA, and the respective protein concentrations of each sample and control was found through the plot of the respective protein curve.

### Status epilepticus

Study mice were pretreated with scopolamine injections intraperitoneally (IP) (1 mg/kg, IP), 30 min prior to status epilepticus induction. Subsequently, Pilocarpine Hydrochloride (280 mg/kg, IP, Sigma Aldrich) was injected and mice were observed over a period of four hours to ensure normal health status. Control mice were injected with equal amounts of sterile saline intraperitoneal. During the first post-pilocarpine observational period (2–4 h after Pilocarpine), mice were evaluated to assess the development of the status epilepticus using the Racine scale^6^. Racine stage equal or above 4 was considered as seizure severity. The mice recovered at least 2 h post-pilocarpine and midazolam (8 mg/kg, IP) was administered as provided by the veterinarian staff at EVMS. Control mice received scopolamine and sterile saline (sham). Mice were left in their appropriate acrylic cages with feed and water over the following 24 h. Mice were monitored every 8 h during the 24-h observational period to ensure healthy levels of hydration and activity.

### AntiCD40L administration

The InVivoMab Anti-Mouse CD40L (CD154) antibody blocks CD40–CD40L interaction in vivo as previously reported in various articles^23,24^. The molecule was validated by BioXCell. For intranasal administration, the InVivoMabAnti-MouseCD40L was diluted to a concentration of 2 mg/mL. The initial concentration was 6.69 mg/mL, approximately 300 µl of antibody solution was diluted into 700 µl of sterile saline. The solution was stored in an Eppendorf tube and kept at 4C until it was to be used. At the time of the experiment, 5 µl of the 2 mg/mL CD40L in sterile saline solution was administered to each naris. Animals were administered InVivoMab Anti-Mouse CD40L (CD154) antibody 2 h before PTZ administration.

### Statistical analysis

CD40 positive cells of different morphologies per field (40X) were semi-quantified. Statistical comparisons were conducted among two groups and standard errors of the mean (SEM) by Student's t-test for statistical significance (*p* < 0.05). Seizures after pilocarpine and percentage of seizure severity was analyzed using non-parametric Wilcoxon signed rank test for statistical significance (*p* < 0.05). All data analyses and graphics were performed using JMP, Version 8, statistical discovery from SAS, www.jmp.com, Cary, NC, 1989–2021.

## Results

### CD40 is expressed in a population of human TLE

**Figure 1:**
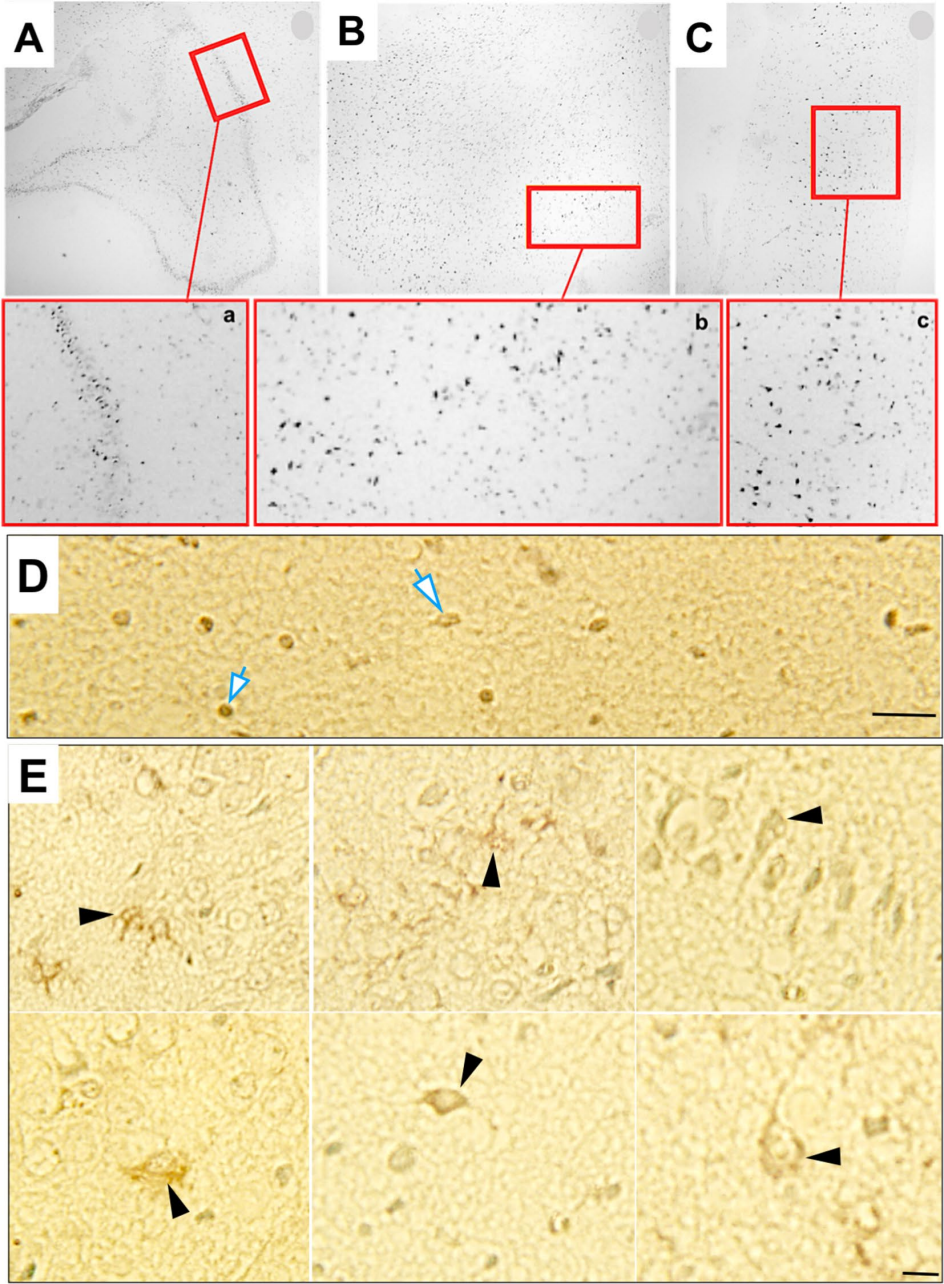
CD40 expression in human Temporal Lobe Epilepsy (TLE): (**A**–**C**) Representative microphotographs from a histological section of human hippocampal regions at low magnification. Inside the red boxes, dentate gyrus (DG) (**A**) and CA3 (**B,C**) from a patient with TLE are present which had been magnified below. These figures depict cellular dispersion and loss of neurons (**A**), disruption of cellular alignment, and loss of neurons (**B,C**). These pathological features are representative of TLE type I- II. (**a–c**) represent magnification of (**A**–**C**). (**D–E**) Representative cellular CD40 IR in TLE. Bar: 100 um.

A public human tissue database shows that the CD40IR is not detected in normal neural tissue (https://www.proteinatlas.org/ENSG00000101017-CD40/tissue). CD40L–CD40 has not been reported in human epilepsy at the time that this manuscript was submitted. Using an immunohistological approach in hippocampal cryosections obtained from patients (n = 4) that underwent neurosurgical treatment for Temporal Lobe Epilepsy (TLE), the CD40 immunoreactivity (IR) was highly expressed in hippocampal regions (Fig. 1). The CD40 IR labeled cells (Fig. 1D,E) resemble astrocytes and neurons^25,26^. Since no current literature or database shows CD40–CD40L expression in normal human neural tissue, the finding of CD40–CD40L in TLE patients was significant but not further analyzed, other than this descriptive analysis finding.

### CD40–CD40L increased after seizures

**Figure 2:**
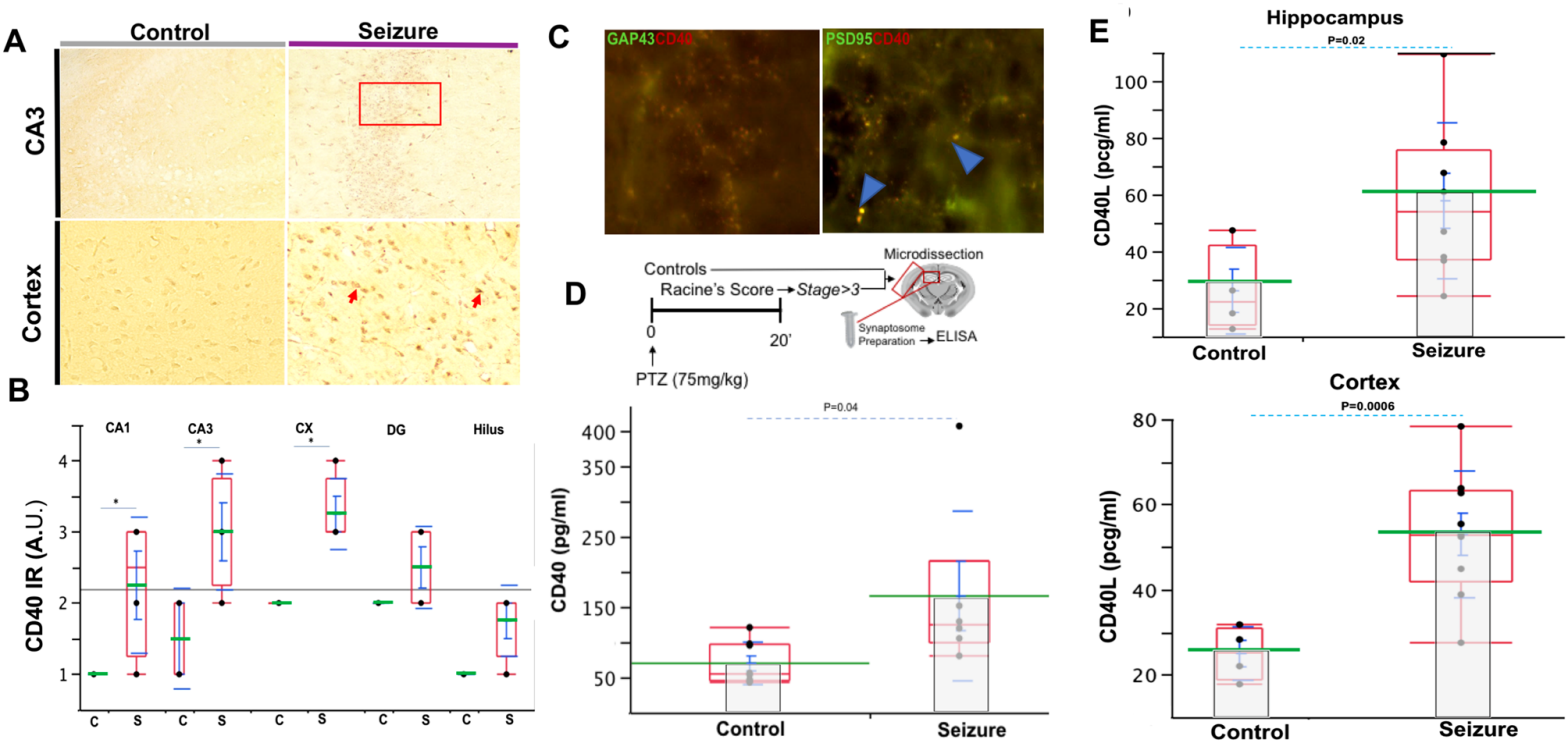
CD40–CD40L increase in the brain after seizure. (**A**) Representative expression of immunoreactivity (IR) against CD40 histological coronal section of CA3 hippocampal subregion and somatosensorial cortex. Note that after a seizure, compared to control, IR increase in neural cells in CA3 and accentuated in cortex respectively. (**B**) Semi-quantitative analysis of IR against CD40 from the histological coronal section of control mice (C) and after seizures (S), (n = 4). CD40 IR is highly expressed in CA1, CA3, and cortex (* = *p* < 0.05). (**C**) CD40 (red) is co-expressed (yellow) with representative presynaptic (GAP-43, green) and postsynaptic (PSD-95, green) terminals in the hippocampus. Blue arrows point to co-expression. (**D**) Experimental design to evaluate concentration of CD40 in synaptosomes. CD40 shows a statistically significant increase in synaptosome in mice with tonic–clonic seizures compare to control (n = 8) vs. Seizure (n = 6) *p* = .049). (**E**) CD40L concentration, measured using ELISA, increase in the hippocampus and cortex after seizures (n = 9) compared to controls (n = 4). Data in graphics shows individual values (black dots), mean (green line); standard error mean (S.E.M., blue bar), Standard deviation (blue line) and quartile (red box).

CD40 is expressed in neurons in the brain from the neocortex and hippocampus in adult mice^27^. However, the hippocampal molecular expression of CD40 is relatively low compared to CD40L^15^. To determine the presence of CD40 in neural terminals by using immunohistological techniques, we observed that the distribution of immunoreactivity (IR) against CD40 presented a trend to be expressed more densely in post-synaptic terminals (Fig. 2C, Supplementary Fig. 2) than pre-synaptic terminals of naive adult mice. Following tonic–clonic seizures (Racine’s score > 3) induced by systemic administration of PTZ, CD40 IR was highly expressed mainly in the neurons located in the somatosensory cortex and as a fibrillar pattern in the hippocampus (Fig. 2A, Supplementary Fig. 2). Moreover, CD40 IR was remarkably increased mainly in CA3 hippocampal subregions and cortex after those seizures compared to control (*p* < 0.05) (Fig. 2B). The concentration of CD40 in synaptosome fractions significantly increased in pooled cortical and hippocampal tissue in mice with tonic–clonic seizures (Racine’s score > 3) (Control: 68.81 ± 10.65 S.EM., n = 8 vs. Seizure: 165.60 ± 49.21 S.E.M., n = 6; *p* = 0.049) (Fig. 2D). However, CD40L in synaptosome fractions did not show a statistically significant difference (data not shown). In addition, CD40 IR was associated with an increase in the concentration of CD40L in the hippocampus (Control: mean 26.10 ± 7.6 S.E.M., n = 4 vs. Seizure: mean 57.77 ± 9.7 S.E.M., n = 8; *p* = 0.02; t = 2.5) and cortex (Control: mean: 24.90 ± 3.13 S.E.M., n = 4 vs. Seizure: mean: 52.93 ± 4.96 S.E.M., n: 9; *p* = 0.0006, t = 2.20) respectively (Fig. 2E). CD40 concentration variabilities were recognized in the hippocampus which could be attributed to cellular expression of the CD40 in differing time points post seizures, despite this an upward trend in post-seizure animals was noted. Future work will investigate the duration and initial activation of CD40 within various cellular populations.

### CD40L–CD40 concentration was higher 24 h after status epilepticus

**Figure 3:**
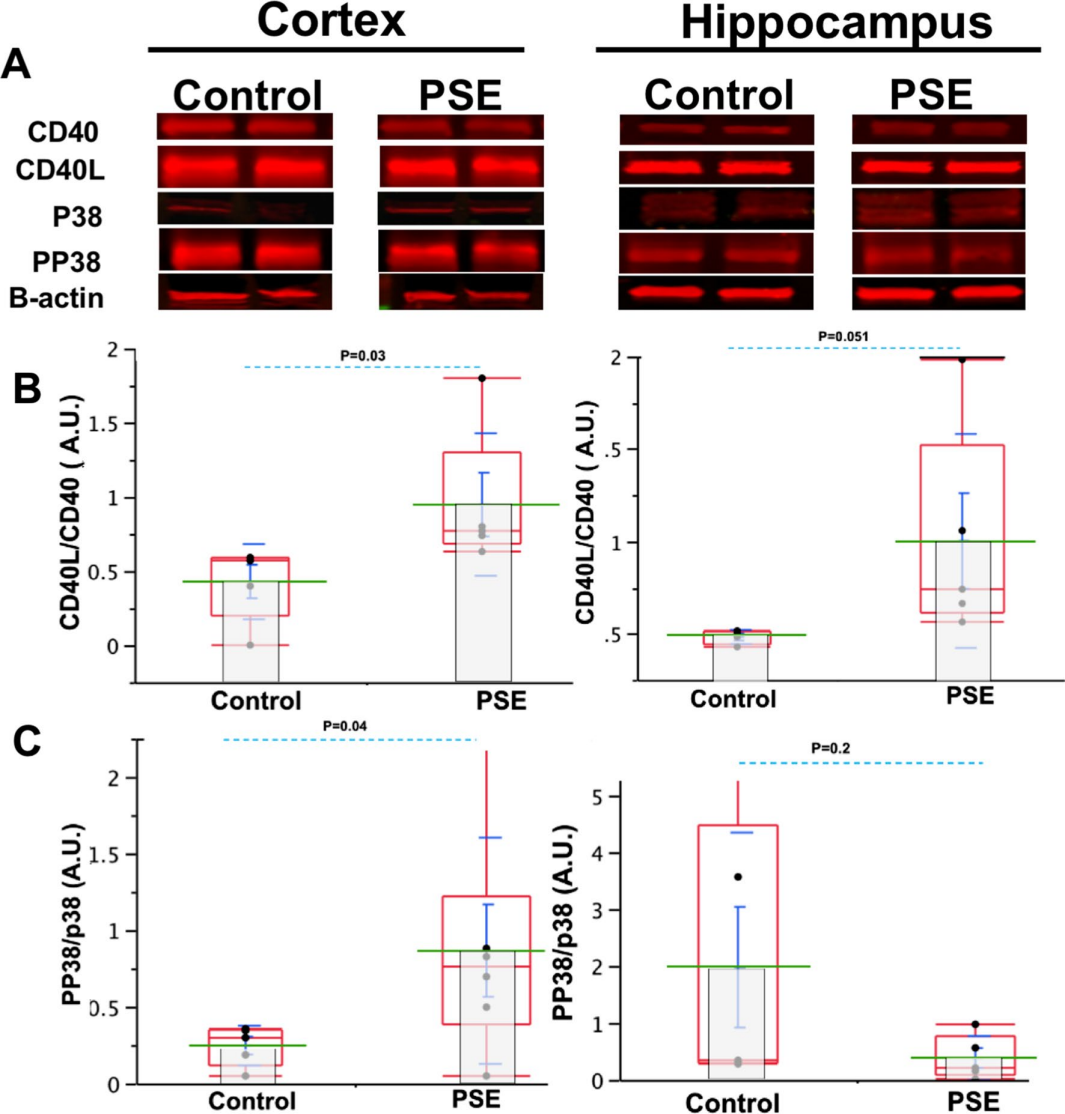
PP38/P38 and CD40–CD40L increases after status epilepticus. (**A**) Representation of bands from respective hippocampus and cortex gels (**B**) CD40L/CD40 expression in the cortex is higher in PSE (n = 5) than control (n = 5) (*p* = 0.03) showing a trend to increase in hippocampus (*p* = 0.051) respectively. (**C**) PP38/P38 expression in the cortex (*p* = 0.04) is higher in PSE than control and non-significant differences between groups are present in hippocampus (*p* = 0.2). Data in graphics show individual values (black dots), mean (green line); standard error mean (S.E.M., blue bar), Standard deviation (blue line) and quartile (red box).

Neuro-inflammation plays a critical role in the development of epilepsy during the acute phase of epileptogenesis, approximately 24 h after SE in the pilocarpine model of TLE^6,9,10^. Using an enzyme-linked immunosorbent assay (ELISA), we observed an increase in both CD40L and CD40 after SE. Consistent with this previous observation, using a Western Blot, it was seen that CD40 increases in the cortex and the hippocampus (Fig. 3B). Considering that CD40L–CD40 interaction is key in activating an inflammatory process, we evaluated the relationship of the concentration of CD40L–CD40 in the brain by analyzing an index between CD40L over CD40 concentrations. We observed that CD40L/CD40 increased relatively more than CD40 in cortex (Control: mean: 0.42 ± 0.1 S.E.M., n = 5 vs. PSE: mean: 0.94 ± 0.21 S.E.M.; n = 5; *p* = 0.03, t = 2.30). CD40L/CD40 also showed an increasing trend in hippocampus (Control: mean: 0.48, ± 0.01 S.E.M.: n = 4 vs. PSE: mean: 1, ± 0.25 S.E.M.: n = 5; *p* = 0.051, t = 2.36) after SE (Fig. 3B). Additionally, since the p38MAPK participates in CD40 signaling pathway and has been implicated in epilepsy via a c-Jun N-terminal kinase^28^, we studied the phosphorylation of p38 after SE^28,29^. During the acute phase of epileptogenesis, the relationship between pp38 and p38 increased in cortex (Control: mean: 0.24 ± 0.12 S.E.M., n = 5 vs. PSE: mean: 0.801 ± 0.7 S.E.M., n = 6; *p* = 0.04, t = 2.62) and a decreasing trend in the hippocampus (Control: mean: 1.9 ± 1 S.E.M., n = 5, vs. PSE: mean: 0.3 ± 0.1 S.E.M., n = 5; *p* = 0.2 ; t = 2.3) (Fig. 3A–C).

### CD40 deficiency attenuated seizure susceptibility

**Figure 4:**
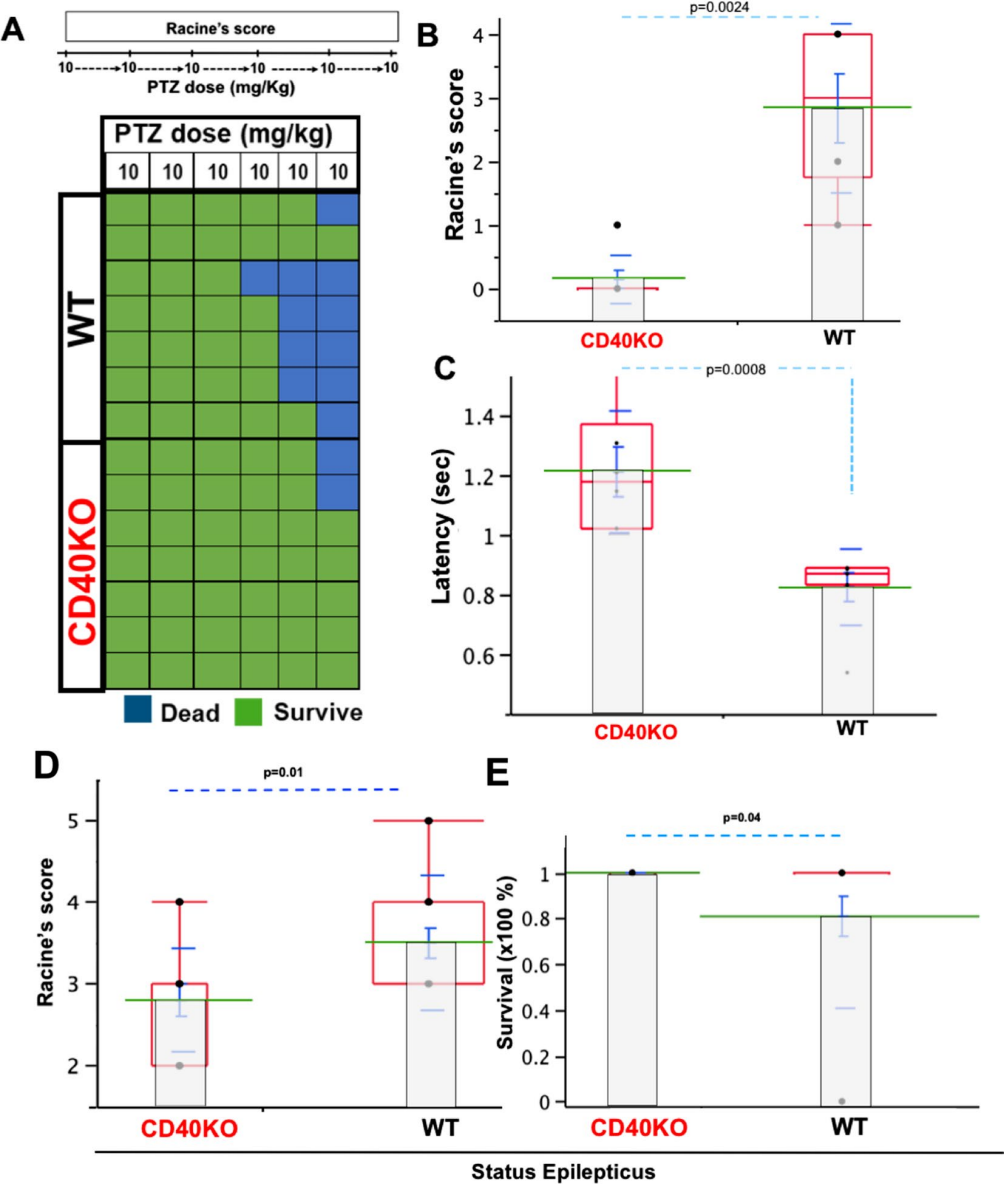
CD40 deficiency lessens seizure susceptibility and reduces mortality. (**A**) Overall mortality/total CD40KO and WT mice respectively. Note lower number of CD40KO dead mice (0.2) compared to WT (0.7). (**B**) CD40KO mice present a remarked reduction of seizure severity at 40 mg/kg doses compared to WT mice, (CD40KO, Racine’s score: 0.1 ± 0.14 SEM; WT: Racine’s score: 2.8 ± 0.54 SEM; *p* = 0.0024). (**C**) CD40KO reduced latency compared to WT mice (CD40KO: 4.45 min ± 0.51 S.E.M vs. WT: 1.09 min ± 0.005 S.E.M; *p* = 0.0008. (**D**) CD40KO mice present lower Racine’s score compared to WT mice during SE induced by pilocarpine; CD40KO (n = 10) vs. WT (n = 21); (*p* = 0.01), (**E**) CD40KO mice show 100% survival 24 h after SE, (*p* = 0.04). Data in graphics shows individual values (black dots), mean (green line); standard error mean (S.E.M., blue bar), Standard deviation (blue line) and quartile (red box).

Given the lack of data concerning the role of CD40 during seizures, we aimed to determine if CD40 deficiency (i.e.CD40KO) affects seizure susceptibility and/or severity. Successive sub-convulsive doses of PTZ (10 mg/kg) were administered to determine the threshold for different types of seizures using Racine’s score^6,25^. CD40KO (n = 7) mice exhibited low mortality (Fig. 4A) and reduced seizure occurrence compared to WT (n = 7), with statistically significant differences at 40 mg/kg (Racine’s score in CD40KO; 0.1 ± 0.14 S.E.M. vs. WT: 2.8 ± 0.54 S.E.M.; *p* = 0.0024; z = 3) (Fig. 4B). In addition, CD40KO demonstrated increased latency in seizure onset at 50 mg/kg compared with WT (CD40KO: 4.45 min ± 0.51 S.E.M. vs. WT:1.09 min ± 0.005 S.E.M.; *p* = 0.0008, t = 2.2) (Fig. 4C).

### CD40 deficiency limited severity of SE

Since the deficiency of CD40 limited seizure susceptibility (Fig. 4), we inquired whether CD40 deficiency was sufficient to mitigate the severity of status epilepticus. We observed that CD40KO mice exhibited seizures with reduced severity compared to WT (Racine’s score in CD40KO: 2.8 ± 0.2 S.E.M., n = 10 vs. WT: 3.42 ± 0.18 S.E.M., n = 21; *p* = 0.01, t = 2.4) (Fig. 4D). Also, CD40 deficiency prevented mortality following SE. (CD40KO: 100% survival vs. WT: 80%, *p* = 0.04, Chisquare = 2.1) (Fig. 4E).

### Anti-CD40L limits seizure susceptibility and severity

**Figure 5:**
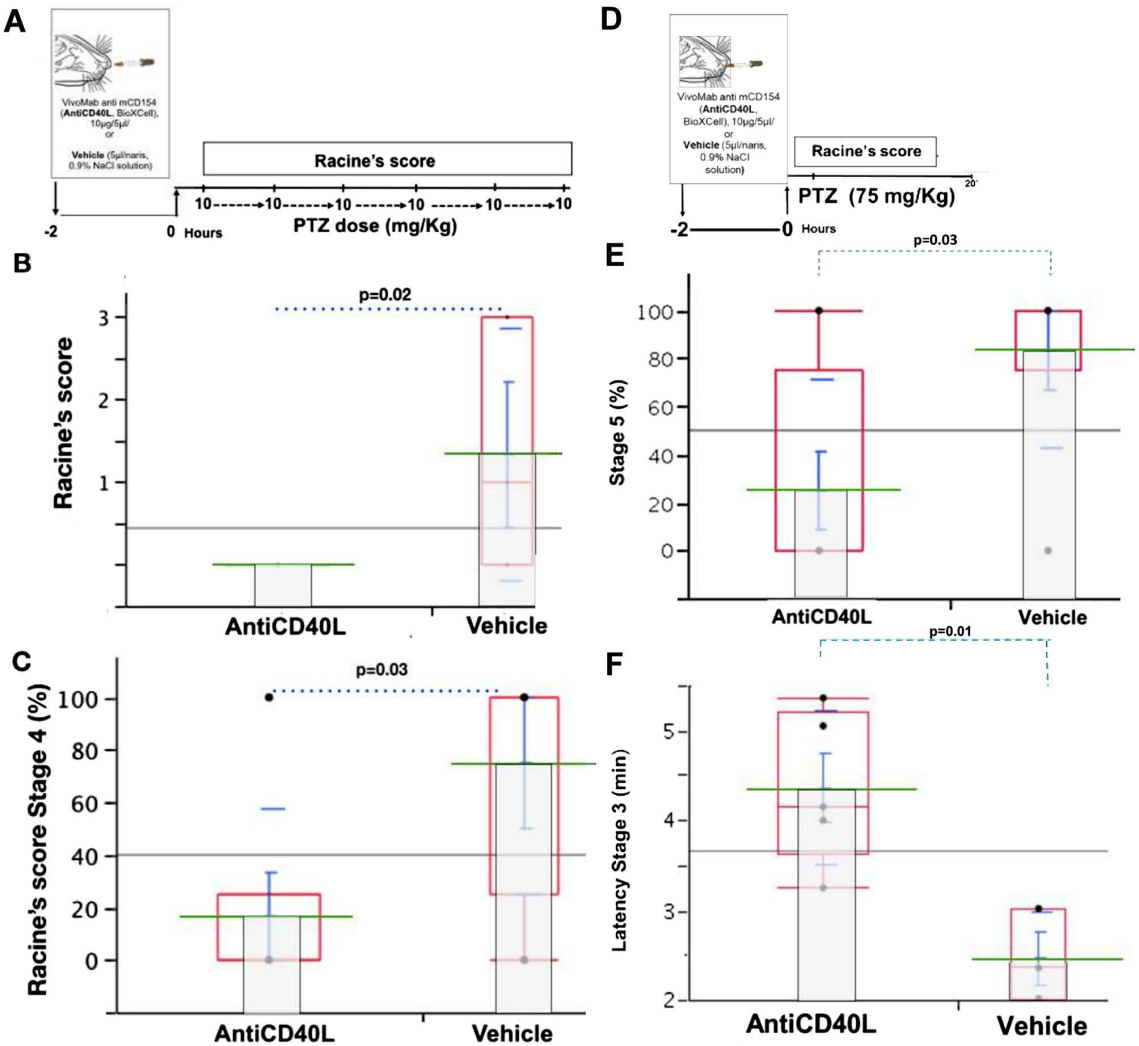
Intranasal administration of anti-mouse CD40L limits seizure susceptibility and seizure severity. (**A**) Experimental design to test anti-CD40 in an in vivo model of seizure susceptibility. Anti-CD40 (BioXCell InVivoMAb anti-mouse CD40L (CD154) or vehicle (sterile saline) was administered intranasally two hours before seizure induction with PTZ. (**B**) Anti-CD40L (n = 6) administration reduces Racine’s score compared to vehicle (n = 4) at 1st, 2nd, and 4th dose of PTZ (*p* = 0.02). (**C**) Anti-CD40L administration decreases the severity of seizures compared to vehicle treatment, as measured by Racine’s score (*p* = 0.03). (**D**) Experimental design to test anti-CD40 within in vivo model of severe seizures. Anti-CD40 ((BioXCell InVivoMAb anti-mouse CD40L (CD154)) or vehicle (sterile saline) were administered intranasally two hours before seizure induction with PTZ. (**E**) AntiCD40L reduces stage 5 seizure severity compared to vehicle. (**F**) AntiCD40L increases latency for stage 3 seizure in AntiCD40 (n = 8) compared to Vehicle (n = 6); *p* = 0.03 and *p* = 0.01. Data in graphics show individual values (black dots), mean (green line); standard error mean (S.E.M., blue bar), Standard deviation (blue line) and quartile (red box).

We hypothesized that the interaction of CD40L–CD40 mediates seizures; therefore, we blocked CD40L–CD40 interaction using inVivoMab Anti-Mouse CD40L (see methods). Anti-CD40 antibody blocks the interaction of CD40L with CD40^23,24^. Since intranasal delivery of anticonvulsive drugs has been postulated in epilepsy and to treat neurodegenerative diseases, an anti-CD40L antibody (see methods) was administered intranasally before seizure induction using PTZ^30–32^. We observed that mice treated with intranasal anti-CD40L antibody exhibited a reduction in seizures at an accumulative dose of 30 mg/kg of PTZ (Racine’s score in Anti-CD40L:0 ± 0 S.E.M., n = 6 vs. Vehicle: 1.8 ± 0.88 S.E.M., n = 4; *p* = 0.02) (Fig. 5B). In addition, anti-CD40L limited seizure severity after respective doses of PTZ (Percentage reaching Racine’s score 4 in anti CD40L:16% ± 16.6 S.E.M. n = 6 vs. Vehicle: 75% ± 25 S.E.M., n = 4; *p* = 0.03) (Fig. 5C). Using a single injection of PTZ at convulsive doses, anti-CD40L antibody-treated animals showed reduction in reaching Racine’s score 5. compared to vehicle (Anti-CD40L:25% ± 16.36 S.E.M. n = 8 vs. Vehicle: 83.33% ± 16.66 S.E.M., n = 6; *p* = 0.03) (Fig. 5E) and a significantly increased latency to reach Racine’s score 3 (Minutes in anti CD40L 4.36 ± 0.37 S.E.M., n = 8 vs. Vehicle: 2.4 ± 0.2 S.E.M., n = 6; *p* = 0.01, t = 1.7) (Fig. 5F).

## Discussion

We observed that CD40 is expressed in human TLE, upregulated after seizures in hippocampal regions, and corresponds with an upregulation of CD40L. In mice, CD40L/CD40 increases 24 h after SE in the cortex, as well as in the hippocampus. A decrease in CD40, whether through CD40- deficient mice or by blocking CD40 interactions using in vivo anti-CD40L antibody administration, limits PTZ- induced seizure severity. CD40 is expressed in 50% of patients with TLE. Due to the lack of CD40 research in the normal brain (https://www.proteinatlas.org/ENSG00000101017-CD40/tissue), we hypothesize that CD40 could play a role in seizure severity or frequency caused by an unresolved neuro-inflammatory process in those patients. We plan to continue to explore this relationship in the future.

It is well-known that CD40 is seen in post-injury inflammation and can be used as a biomarker. This raises the question of the role of CD40 in epileptogenesis^33^. Levels of CD40 were increased predominantly in neural terminals in the hippocampus and cortex following induction of seizures. An increase in CD40L was also seen in the hippocampus and cortex, 24 h after status epilepticus, indicating a possible sustained response. However, additional studies are needed to verify the CD40L–CD40 post-seizure time course, its relationship to various brain regions, and cellular population response. The results of these findings will determine if levels of CD40L–CD40 is an acute response, sustained after seizures, or spontaneously resolved to physiological levels after seizure culmination.

The CD40KO mice, demonstrated a statistically significant reduction in seizure susceptibility, suggesting that CD40–CD40L may be directly involved in mediating ictogenesis or as a mechanism of seizure propagation. The CD40KO mice that developed seizures had a statistically significant decrease in seizure severity with no mortality, suggesting that CD40 mediates a mechanism with neuronal hyper-excitability. These observations warrant further exploration of the role of CD40 neurotransmission impairments in epilepsy in depth. Recently, studies show that postnatal CD40-deficient mice present with a reduction of excitatory hippocampal pyramidal terminals neurons compared to wild type littermate mice. At this location, dendrite arbors of inhibitory striatal medium spiny neurons are increased in size and more branched in the absence of CD40^15^. Additional research is required to evaluate how CD40 mediates the formation of aberrant terminals in epileptogenesis^6^. Anti-CD40 attenuates the release of these cytokines and the resultant blood–brain barrier (BBB) dysfunction^34^. Several studies have implicated CD40–CD40L interaction in the upregulation of endothelium adhesion molecules and leukocyte transmigration, which may influence the integrity of the BBB^26,27^. Further BBB integrity and PTZ pharmacodynamics studies might be useful in our understanding of this scenario.

Our data show that intranasal administration of in vivo anti-CD40L antibody is effective to limit acute seizures (Fig. 5). Anti-CD40L antibodies are currently one of the leading new treatments still under investigation in patients with cancer due to their ability to modulate immune responses. The mechanism of anti-CD40L antibodies in cancer therapy is to enhance the immune system through the downstream effects of CD40 activation and marking malignant cells for destruction^35^. Limited analysis has been conducted on anti-CD40 as a receptor antagonist/blocker. In this condition, our approach could be useful to explore a GBM induced seizure.

In addition, the CD40 molecular signaling may play a role in the development of epilepsy and drug-resistant epilepsy through the activation of P38 MAPK. Our data reveals an increase in phosphorylated p38 in our mouse models with epilepsy. This is consistent with previously published literature showing that phosphorylation of p38MAPK may contribute to epileptogenesis either directly or through downstream effects^27^.

CD40 is commonly expressed on immune cells such as monocytes and dendritic cells and non-immunological cells such as neurons, microglia, and endothelial cells^33,36,37^. Additionally, CD40 contributes to the post-injury inflammatory environment^33^. Microglial activation by lipopolysaccharides has been shown to increase the expression of CD40 and CD40L secretion, in turn increasing the secretion of the pro-inflammatory cytokines TNF–α, IL-1β and IL-6^34^.

On the other hand, CD40 could mimic or act synergistically with the function of the pro-inflammatory cytokine TNF-α which plays a role in acute seizures^38^. TNF-α, released from physiologically activated microglia and astrocytes, contributes to the homeostatic level of glutamate via TNF receptor 1 (TNFR1) and regulates the formation and organization of excitatory and inhibitory synapses^39–41^. Following an injury, TNF-α up-regulates AMPA receptors, augmenting glutamatergic transmission causing neurotoxicity and hyper-excitability exacerbated by induction of GABA receptor endocytosis, which reduces the inhibitory drive. Since TNF signaling may have an important role not only in ictogenesis, but also in early phases of epileptogenesis, anti-TNF-α therapy for epilepsy is also under debate due to the suspected risks of demyelination, infection, and cancer development.

Although there is an increase in CD40L in epilepsy, its role in TLE has not been fully elucidated, and the present study suggests that CD40L–CD40 interaction could be a promising target for early therapeutic intervention in TLE and could prevent the onset of TLE. This approach could also be expanded to neurological disorders that similarly involve disruption of neuronal networks, such as Alzheimer’s disease. The results described in this study highlight the involvement of the CD40–CD40L pathway in the development of epilepsy. This provides the groundwork for potential exploration of CD40–CD40L as a molecular target for the prevention and treatment of epilepsy.

## Supplementary Information

Supplementary Information.

## Data Availability

IACUC protocol #17-012.
