# Author Correction: Downregulation of CD40L–CD40 attenuates seizure susceptibility and severity of seizures

**DOI:** 10.1038/s41598-021-02005-8

**Published:** 2021-11-25

**Authors:** Esther Pototskiy, Katherine Vinokuroff, Andrew Ojeda, C. Kendall Major, Deepak Sharma, Taylor Anderson, Kendall Howard, Ronen Borenstein, Alberto E. Musto

**Affiliations:** 1 Department of Pathology and Anatomy, Eastern Virginia Medical School, 700 W. Olney Road, Lewis Hall, Office 2174, Norfolk, VA 23507 USA; 2 Department of Neurology, Eastern Virginia Medical School, 700 W. Olney Road, Lewis Hall, Office 2174, Norfolk, VA 23507 USA; 3 Eastern Virginia Medical School, Norfolk, VA USA; 4 Department of Microbiology and Molecular Cell Biology, Eastern Virginia Medical School, Norfolk, VA 23507 USA

Correction to: *Scientific Reports* https://doi.org/10.1038/s41598-021-96760-3, published online 26 August 2021

Ronen Borenstein was omitted from the author list in the original version of this Article.

The Author Contributions section now reads:

A.E.M.: conceived the idea, performed experiments, analyzed data, wrote and reviewed the manuscript, A.A.O., C.K.M., T.A., E.P., K.V., K.H., S.D, RB.: performed experiments, analyzed data, wrote and reviewed the manuscript.

The original Article has been corrected.

