# Supplementary material for: Downregulation of CD40L–CD40 attenuates seizure susceptibility and severity of seizures
Source: Sci Rep. 2021 Aug 26;11:17262. doi: 10.1038/s41598-021-96760-3 (PMC8390750; doi:10.1038/s41598-021-96760-3)

# **Downregulation of CD40L-CD40 attenuates seizure susceptibility and severity of seizures**

Esther Pototskiy #^3,4^, Katherine Vinokuroff ^3,4^, Andrew Ojeda^1^ , C. Kendall Major^3^, Sharma Deepak^3^, Taylor Anderson^1^, Kendall Howard^3^, Ronen Borenstein^5^ and Alberto E. Musto^1.2^*,

1 Department of Pathology and Anatomy, Eastern Virginia Medical School, Norfolk, Virginia, USA.

2 Department of Neurology, Eastern Virginia Medical School, Norfolk, Virginia, USA.

3 Eastern Virginia Medical School, Norfolk, VA

4 Biomedical Sciences Old Dominion University, Norfolk, VA

5 Department of Microbiology and Molecular Cell Biology, Eastern Virginia Medical School, Norfolk, Virginia, USA.

*To whom correspondence should be addressed:

Alberto E. Musto M.D., Ph.D., F.A.E.S.

Department of Pathology and Anatomy

Department of Neurology

Eastern Virginia Medical School

700 W. Olney Road

Lewis Hall, Office 2174

Norfolk, Virginia 23507

TE: 757-446-5774

[Email: mustoae@evms.edu](mailto:Email%3Amustoae@evms.edu)

**Supplementary information:**

**Materials and methods are described in the body of the manuscript.**

**Figure legends:**

**Supplementary Figure 1:** Immunofluorescence of PSD-95, CD40, and GAP-43 in hippocampus, arrows pointing to expression of GAP-43, CD40, and PSD-95

**Supplementary Figure 2: (A)** Difference of immunoreactivity in cortical region from different mice: CD40 in knockout (CD40KO), WT control, WT Racine’s Score Stage 1 and Stage 4 seizure. **(B)** Presence of CD40 expression in different brain regions: Cortex, CA1, CA3, and Dentate Gyrus regions of the hippocampus. Bar 100µm.

**Supplementary Figure 3:** Representative western blots imaging with labeled samples.

Supplementary Figure 1


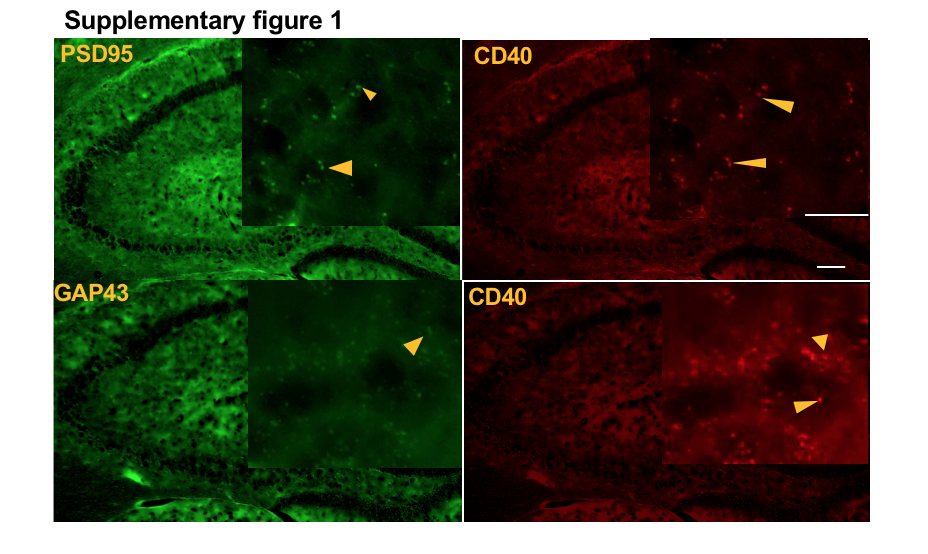


Supplementary Figure 2


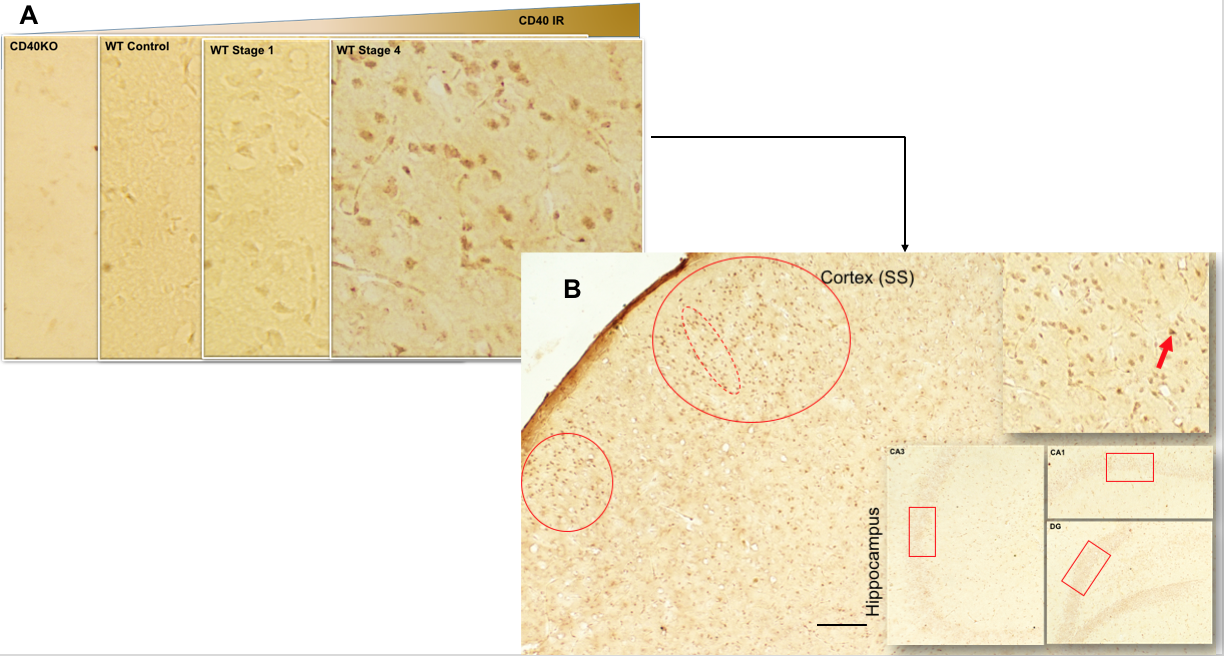


Supplementary Figure 3


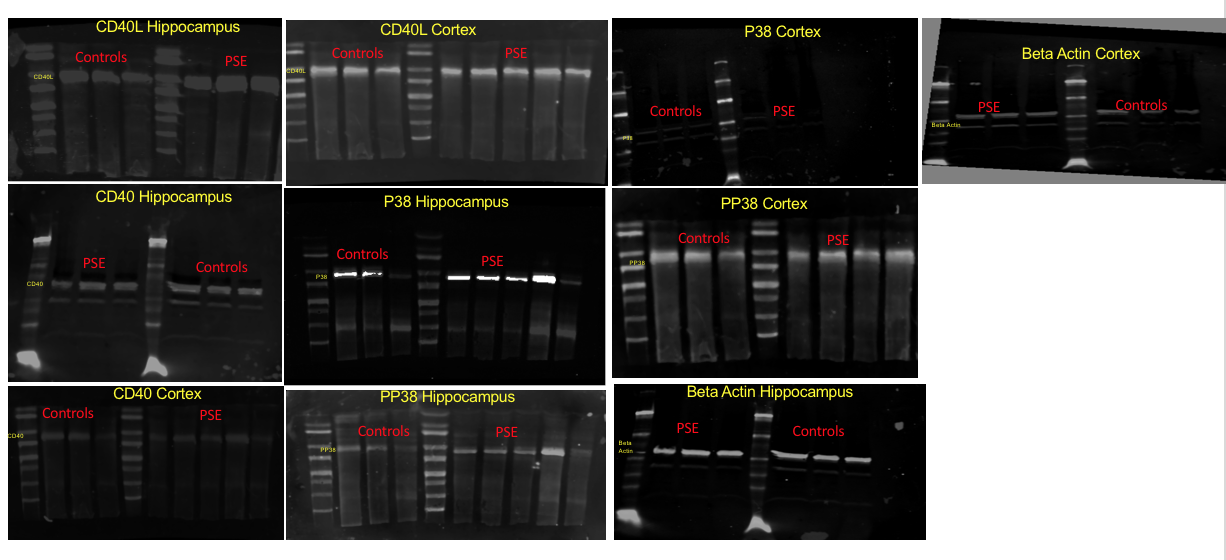

Supplement: Supplementary file 1 — Supplementary Information. [file 41598_2021_96760_MOESM1_ESM.docx]
